# Assessing fitness-to-practice of overseas-trained health practitioners by Australian registration & accreditation bodies

**DOI:** 10.1186/1472-6920-12-91

**Published:** 2012-09-29

**Authors:** Brett Vaughan, Vivienne Sullivan, Cameron Gosling, Patrick McLaughlin, Gary Fryer, Margaret Wolff, Roger Gabb

**Affiliations:** 1 Osteopathy Unit, School of Biomedical & Health Sciences, Victoria University, Melbourne, Australia; 2 Institute of Sport, Exercise and Active Living, Victoria University, Melbourne, Australia; 3 Department of Epidemiology & Preventive Medicine, Monash University, Melbourne, Australia; 4 British School of Osteopathy, London, United Kingdom; 5 Teaching & Learning Taskforce, Faculty of Health, Engineering & Science, Victoria University, Melbourne, Australia

## Abstract

**Background:**

Assessment of fitness-to-practice of health professionals trained overseas and who wish to practice in Australia is undertaken by a range of organisations. These organisations conduct assessments using a range of methods. However there is very little published about how these organisations conduct their assessments. The purpose of the current paper is to investigate the methods of assessment used by these organisations and the issues associated with conducting these assessments.

**Methods:**

A series of semi-structured interviews was undertaken with a variety of organisations who undertake assessments of overseas-trained health professionals who wish to practice in Australia. Content analysis of the interviews was used to identify themes and patterns.

**Results:**

Four themes were generated from the content analysis of the interviews: (1) assessing; (2) process; (3) examiners; and (4) cost-efficiency. The themes were interconnected and each theme also had a number of sub-themes.

**Conclusions:**

The organisations who participated in the present study used a range of assessment methods to assess overseas trained health professionals. These organisations also highlighted a number of issues, particularly related to examiners and process issues, pre- and post-assessment. Organisations demonstrated an appreciation for ongoing review of their assessment processes and incorporating evidence from the literature to inform their processes and assessment development.

## Background

Assessment of fitness-to-practice in a jurisdiction is commonplace where a person trained in one country wishes to practice in another. These assessments take many forms and are designed to assess the competency or the capability of the practitioner. The overarching role of the assessment is to protect the public from practitioners who are not competent [1-3].

In Australia, the assessment of overseas trained health professionals who wish to practice in Australia (and in some cases New Zealand) rests with the health professional accreditation bodies or professional associations. In the case of the professional accreditation bodies, this role is assigned by regulators under the Health Practitioner Regulation National Law Act (2009) [4]. Each accreditation body is also often charged with the responsibility of assessing the suitability of pre-registration university programs for that profession.

The assessments undertaken by these bodies varies depending on the competencies and capabilities set out for that profession, with the methods of assessment chosen to ensure that a range of these competencies and capabilities are assessed [3,5], often in multiple ways. It may be however, that the assessments measure those competencies or capabilities that are easily assessed, and omit those that are not [6]. The purpose of these assessments is to protect patients. Therefore, the methods chosen to assess candidates should be reliable, valid, feasible and acceptable [7,8]. In this way patients are exposed only to practitioners deemed competent to practice in that profession. In addition, it is also important that assessment methods are continuously reviewed as part of quality assurance processes [3].

Whilst very little has been published on the methods of assessment [8] and issues surrounding the assessment of overseas trained health practitioners in Australia, there has been some discussion of the political and workforce issues (e.g., complex procedures, direct and indirect discrimination, poor provision of information) surrounding international medical graduates wishing to practice in Australia [9-13]. There are examples throughout the literature of assessment methods in licensing exams.

Pharmacists seeking to practice in Ontario, Canada undertake a Prior Learning Assessment (PLA) that assesses the candidates learning through both formal and informal education [14]. The PLA used in this context is both an assessment of transcripts, portfolios etc. and performance in an Objective Structures Clinical Examination (OSCE).

The OSCE format is used widely in fitness-to-practice assessments. Austin et al. [15] have described the development of the OSCE for pharmacy graduates with Munoz et al. [16] presenting data on the reliability, validity and generalisability of the examination. Austin et al. [14] suggests that standardised English-language proficiency tests (e.g. IELTS) may not be appropriate for cultural competency and communication skills required for pharmacy practice, even though this is a particularly important criteria where there is diversity in the candiates’ English language proficiency [17].

This suggests that assessment of English language proficiency should form part of the assessment process, and organisations should not rely solely on standardised English-language tests. In addition to English-language assessment, Archer [18] suggests that assessment of psychosocial skills form part of a licensing examination. These communication and psychosocial skill issues are quite relevant, as Tamblyn et al. [19] have demonstrated that patient-practitioner communication and clinical decision making during fitness-to-practice assessments correlate with complaints to professional regulation bodies.

The aim of the current paper is to identify the methods of assessment used by those bodies that undertake the assessment of overseas trained health professionals in Australia, and to identify the issues surrounding these assessments and how these issues are managed.

## Methods

### Study design

Semi-structured interviews were used to explore how Australian health professional assessment bodies assess overseas-trained practitioners. An interview schedule (Table 1) was developed based on the findings of a systematic search and critical review of the health professional assessment literature and preliminary information collected from the websites of these bodies. A semi-structured format was chosen so that information could be gathered on specific areas of interest (e.g., structure of assessment framework) while still providing participants with the opportunity to describe their unique experiences associated with assessment.

**Table 1:** Interview schedule

	
1. How do you determine the initial eligibility of an overseas trained practitioner to undertake further assessment? Why?	
2. How does your organisation assess:	
a. Basic sciences?	
b. Taking a clinical history?	
c. Doing a clinical assessment?	
d. Diagnosis & clinical reasoning?	
e. Treatment?	
f. Aftercare and follow-up?	
g. Practitioner-client (osteopath-patient) communication.	
h. Commitment to continuous improvement and professional development?	
i. Knowledge of the Australian health system?	
j. Collaboration with other health professionals?	
3. How does your organisation set the standard (pass mark) for each assessment task?	
4. How does your organisation select assessors/examiners? Do you require assessors/examiners to undergo training?	
5. How does your organisation review the performance of your assessment processes for overseas trained practitioners?	
6. What do you believe are the strengths of your organisation’s assessment processes for overseas trained practitioners?	
7. What do you believe are the weaknesses of your organisation’s assessment processes for overseas trained practitioners?	
8. How do you manage risks to the public and to the profession in the assessment of overseas trained practitioners?	
9. Does your organisation use supervised practice as part of the assessment of overseas trained practitioners?	
10. How does your organisation deal with candidates who fail part of the assessment process?	
11. How cost-efficient are your organisation’s processes for assessing overseas trained practitioners? How significant are the expenditure and the income associated with the assessment of overseas trained practitioners in your organisation’s annual budget?	
12. Does your organisation automatically recognise graduates from any overseas training programs?	

The study was approved by the Victoria University Faculty of Health, Engineering and Science Human Research Ethics Committee.

### Participants

Thirteen (N=13) professional bodies who assess fitness-to-practice of overseas trained health professionals were approached to participate in interviews exploring current practices in the assessment of overseas-trained health professionals wishing to practice in Australia.

The interviews were conducted by a researcher (VS) experienced in conducting interviews for research.

### Data collection

All interviews were audio-recorded and transcribed verbatim. Notes were taken during interviews to include any relevant non-verbal cues and to assist with data transcription (e.g., when the quality of the recording was compromised by background noise). Participants were sent a copy of their transcribed interviews and were asked to make any necessary changes (e.g., if the researcher had misheard a statement) and/or add any additional comments.

### Data analysis

Utilising NVivo (QSR International, Victoria, Australia) a primarily directed approach to content analysis was used to select and focus data from transcriptions and notes [20,21]. Based on previous research [20,22] some themes were set prior to conducting interviews allowing for semi-structured guidelines to be developed. Nonetheless, as the research was also investigating a relatively sparse area of research, a conventional or inductive content analysis approach was also used to identify any additional themes and categories that emerged [22,23].

## Results

Eleven (N=11) organisations agreed to participate (Table 2) with one or two representatives taking part in each interview. Representatives of the organisations were the chief executives and/or those in charge of the development, implementation and conduct of the assessment process.

**Table 2:** Participating organisations

	
1. Australian Dental Council	
2. Australian Institute of Radiography	
3. Australian Medical Council	
4. Australian Nursing and Midwifery Council	
5. Australian Pharmacy Council	
6. Australian Physiotherapy Council	
7. Australian Podiatry Council	
8. Australian Psychological Society	
9. Council on Chiropractic Education Australasia	
10. Optometry Council of Australia and New Zealand	
11. Speech Pathology Australia	

By paying particular attention to patterns, regularities, irregularities and propositions within the data [21,24,25] four interconnected categories were generated from the interview data with each theme also generating first- and second-order themes.

### Assessing (Theme 1)

**Figure 1:**
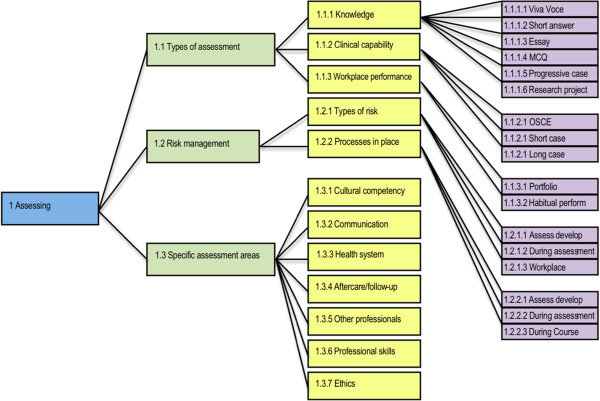
Sub-themes identified within the Assessing theme.

Professional bodies used a variety of assessment strategies to assess whether an overseas-trained practitioner was eligible to be registered to practice in Australia (Figure 1).

*Desktop assessment* (Theme 1.1.1.1) was used in two ways. In the first instance bodies used the assessment as an initial step in the process where candidates were required to provide evidence and information regarding their eligibility to take part in the assessment process. For some bodies the desktop assessment was the sole assessment tool. Assessing a candidate’s training was identified as a very important step:

"…because can’t assess everythingthat person needs, shouldbe knowing as apractitioner, you have torely on their trainingto have given themsome of it. Ijust don’t think thatcompetence-only assessment is eitheraffordable or realistic"

Although a traditional unstructured *Viva voce* (Theme 1.1.1.2) or oral examination was not common practice, it was not unusual for clinical examinations to include some form of verbal questioning to assess candidate’s clinical reasoning or to assess performance criteria that may not have been covered as part of the clinical examination.

*Short-answer* (Theme 1.1.1.3) questions were predominantly used in conjunction with multiple choice question (MCQ) assessments and were often based on clinical scenarios. The *Essay* (Theme 1.1.1.4) or long-answer question was used infrequently and only as part of a multi-format written assessment. An *MCQ* (Theme 1.1.1.5) examination was commonly used as the first step in the assessment process to assess theoretical, basic science and/or clinical knowledge. Vignettes or situational questions were often used rather than those assessing knowledge of facts as this method had been found to be better at assessing areas such as clinical reasoning, judgement and diagnosis. This type of question was also reported to be more discriminating than other types of question:

"When it was factualrecall they were scoringaround 40% plus correct,soon as you putin a vignette theydropped below 25%."

*Clinical capability* (Theme 1.1.2) was assessed using different methods. The *OSCE* (Theme 1.1.2.1) was often used as a method of assessing clinical skills (e.g. history taking, after care) and clinical reasoning. The stations did not necessarily use real or standardised patients. The *Long-case* (Theme 1.1.2.2) was commonly used to assess the performance of clinical skills. Patient selection strategies ranged from a purposeful selection of patients based on age and/or medical condition to accepting walk-in patients.

Risk management was a primary concern for most organisations, particularly for those professions where the potential risk to patients was high. Issues and solutions were identified from a variety of aspects including minimising risk to the community from practising health professionals, decreasing the risk of harm to those participating in examinations, reducing harm to the candidate by placing them in a situation they are ill-equipped to handle, considering the candidate’s potential impact on colleagues and the risk of candidate’s appealing or engaging in legal action because of the assessment outcome:

"… however it isdone at the endof the day ifyou are putting yourstamp on them youhave to know thatthey can deliver, anddo no harm."

During *assessment* (Theme 1.2.2.2), the main risk-management processes risks were identified as those associated with stringency of evidence confirmation, vigilance when assessing areas where harm can be caused, requiring a demonstration of clinical skills and competency, training assessors in policy and process to follow if model/patient is at risk, transparency to candidate in terms of safety performance indicators, policy and procedures and running examinations well using good staff.

The assessment of cultural competency was increasingly important for most professional bodies and was seen as a complex issue to assess:

"… the cultural competencehas become more ofan issue … beyondcommunication you have tohave an understanding ofthe culture of theindividual you are dealingwith and I don’tthink we are atthat stage yet …"

The main focus identified in the area of cultural competency was the ability of candidates to treat patients from culturally and linguistically diverse backgrounds, including indigenous Australians and/or patients of different ages. Cultural competency was sometimes assessed in written and clinical examinations by presenting scenarios that included cultural aspects. In examinations where real patients were included, most organisations screened the patients and did not include those with language difficulties that required an interpreter.

Another issue raised in terms of cultural competency was the possibility of culturally-influenced responses. Caution was advised when developing assessments so that candidates from different cultures are not mis-cued. Concern was also expressed about the inability of candidates to gain employment without an understanding of the Australian workplace culture (e.g., allied health support staff), particularly if competing against Australian candidates for positions.

Assessing a *candidate’s communication skills* (Theme 1.3.2) could include their interaction with patients, other professionals, patients’ families and particular professional environments. Some respondents noted that communication was assessed throughout the examination process, as without adequate communication skills the candidate could not complete the required tasks. Others specifically assessed communication skills, including building rapport, listening skills and sensitivity to client and information gathered.

For some professional bodies communication was seen as ‘…stuff that is not easy for us to assess’. One concern was that written assessments (e.g., portfolios, MCQs) or simulation-style assessments did not allow observation of candidates’ communication skills in areas such as relationship-building with the client. One strategy to overcome this in portfolio assessment was to ask the candidates to submit a video of a treatment session or consultation. Another solution identified was a change to workplace assessment.

Knowledge of the *Australian health system* (Theme 1.33) was considered from two perspectives. The first was the effect on patients and colleagues resulting from a practitioner’s lack of knowledge of the system. The second was the effect on the candidate’s performance in assessment tasks due to their lack of knowledge. Of particular concern were areas such as occupational health and safety, ethical issues and systems and processes. One problem identified was differences between the states

"… you are constructingthose questions you thenfelt the problems betweenthe states, and thosedifferences you might thinkyou have got aterrific question and thensomeone from SA [SouthAustralia] will say noit’s totally different inNSW [New South Wales].That is where youend up with abig problem."

One solution suggested was a consensus between the states or a national standard. Although some organisations did not directly assess knowledge of the Australian health system they provided information on the system to candidates through publications or guest speakers. Another recommendation for candidates was to spend some time observing in an Australian clinical setting. This was seen as a very effective learning experience for candidates who were struggling in the area.

### Processing (Theme 2)

**Figure 2:**
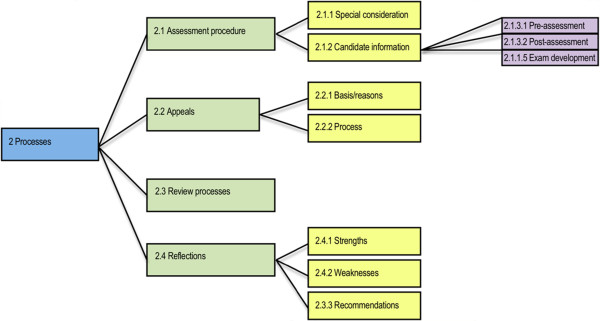
Sub-themes identified within the Processing theme.

For each organisation the assessment of overseas-trained professionals was a process that was continually being reviewed with the aim of increasing its efficiency and effectiveness (Figure 2).

Decisions about a candidate’s eligibility to participate in the assessment process were based on a number of criteria. Satisfactory completion of *Courses and qualifications* (Theme 2.1.1.1) was a common criterion. Prior to entry to any examination, most organisations required candidates to demonstrate that they had successfully completed an approved course that was deemed as equivalent to studies in Australia. In some cases courses were required to be approved by specific councils or organisations in the country and in other cases courses from particular countries were deemed acceptable.

Another strategy used was to recognise equivalent *Examination or accreditation systems* (Theme 2.1.1.2) coupled with at least 12 months of clinical practice in a particular environment. Examination and accreditation systems were accredited on the basis of documented quality and similarity in structure and standard to Australian systems.

Professional bodies required candidates to complete *English language* qualifications (Theme 2.1.1.3), either the Occupational English Test (OET) or the International English Language Testing System (IELTS). Candidates were required to, or would be in the near future, gain at least a B in the OET or a 7 on the IELTS in all sections in one sitting.

Several professional bodies conducted their written examination at both on-shore and off-shore locations (Theme 2.1.2). Factors taken into consideration in adopting this strategy were convenience for candidates (i.e., not having to travel to Australia) and cost to the organisation. Several professional bodies that were unable to conduct stand-alone testing sessions due to candidate numbers linked with other Australian professional bodies to run combined sessions. The examinations were conducted through experienced and reliable overseas venues:

"So the offshore isconducted through a clearinghouse … they doall the arrangements withthe off-shore venues forabout 6 professions, becauseyou know we mightonly have one [candidate]in Tehran but theremight be two [omitted– other allied healthprofessionals] and three [omitted– other allied healthprofessionals] so then we’renot all paying forinvigilators and things likethat … I guessthere are venues inmost places around theworld …"

There was some variation in what *Post-assessment* information was provided to candidates about their performance on the assessment (Theme 2.1.3.2). Many professional bodies provided candidates who fail with information on the areas of the assessment they needed to improve in. Some only provided this information if the candidate appealed:

"The advice from DEEWR[Department of Education, Employment& Workplace Relations] isno. Very simple andclear. On appeal weusually do, then explaina bit more clearly.We will say tothem you need todo the following …"

"… [examiner] gives recommendationsto the candidate andthen we pass thoseon … has noproblem with the candidatesringing and talking tothem about the assessmentand asking advice andthings like that …"

One organisation noted that cultural expectations need to be considered in communicating results:

"You know in Australiawe tend to sugar-coatbad news … we’vedone away with it,successful or not successful,or you know, suitableor not suitable, becauseeven though it isnot an examination processas such, it’s anassessment. Culturally, people wantto hear did Ipass or fail?"

*Examination development* (Theme 2.1.4) was a long and costly commitment for professional bodies and required ongoing review. Some strategies of development included use of expert panels, sharing with similar overseas professional bodies and sharing information with Australian educational institutions. Questions were occasionally trialled by being included within scheduled examinations for Australian pre-registration programmes and by practising professionals at events such as conferences. Including trial questions as part of scheduled examinations was generally seen as the most efficient method, as it could be difficult and costly to encourage students and professionals to participate with commitment in trialling questions:

"We thought that payingthem [final year students]and telling them howimportant it was wouldbe enough for themto take it seriouslybut the exam was3 hour duration andwe made them stayfor 1½ hours butwe could tell thatsome of them didn’t- it didn’t reallywork - didn’t giveit a really goodgo."

Even so, using graduating students was seen as desirable as often the passing score for the examination was based on the graduating Australian student level.

The *basis for appeals* (Theme 2.2.2) appeared to be both procedural or related to candidate impairment (e.g., feeling unwell). Some organisations only considered procedural appeals that asserted that the assessment process had been defective. Professional bodies worked hard to minimise the frequency of appeals by creating a comprehensive assessment blueprint linked to professional standards; having transparent processes; following guidelines in areas such as patient selection; and monitoring candidate performance by methods such as videotaping clinical assessments or recording key strokes in computerised examinations.

*Review of assessment processes* (Theme 2.3) tended to be ongoing with assessors encouraged to provide feedback and assessment data analysed after each examination. Some organisations were starting to include an analysis of examiner performance in their review. Another, less common, internal review strategy was to survey candidates on their assessment experience. In most instances reviews were conducted by a committee.

Professional bodies were asked to identify the main strengths (Table 3) and weaknesses (Table 4) of their processes. They were also asked to discuss any changes planned for future implementation or changes they would like to implement (Table 5).

**Table 3:** **Strengths of the assessmentprocesses**

	
· Multiple forms of assessment – fairer for candidates in terms of examination format preferences and assesses candidates on different areas of competency	
· Practical examination – seeing the candidate put theory into practice	
· Examination difficulty –stringent test of eligibility for registration to practice in Australia	
· Multiple examiners – improves fairness of decisions	
· Independent examiners – improves fairness of decisions	
· Consistency across Australia and New Zealand – minimises the possibility of candidates ‘shopping’ for easier assessment	
· Consistency across candidates	
· Consistency across all registration applicants – as all applicants including Australian university graduates, Australian-trained professionals re-entering the profession and overseas-trained professionals have the same basis for assessment there is ‘absolute equity’ and no perception that assessment may be more difficult for overseas applicants	
· Transparent – providing candidates with comprehensive information on the assessment process was seen as beneficial to reduce candidate stress and decrease the chance of candidate complaints	
· Conducted over time – minimises the possible bias from a candidate who has a ‘bad day’	
· Case-by-case assessment – each course and qualification considered can be examined on its merits rather than being rejected because it is not on a pre-approved list	
· Rigorous process – assessment guidelines allow for cross-checking and panel referral for borderline applicants; stringent documentation checking including references; and strict examination monitoring	
· No need to come to Australia – reduces the cost for candidates	
· Onus on candidate to provide evidence – ensures the candidate has had to consider and reflect on the Australian standards to fulfil the assessment criteria	
· Rigorous assessment development – reduces future problems by putting in high levels of effort and resources from the beginning	
· Good and varied staff – enhancing examination development by engaging professionals from a variety of backgrounds who understand the assessment process and do not alienate other examiners	

**Table 4:** **Weakness of the assessmentprocesses**

	
· Consistency across assessment sites – candidates share information on perceived easier assessment and this results in application drift	
· Limited sampling – through snapshot assessments ‘you can only sample a certain amount in what we are looking at whether it’s the skills, domains, the presenting clinical conditions, whatever’	
· Availability of venues – limited availability of high demand venues affects the efficient running of the examination	
· Recruitment of real patients – although patients are recruited in advance not all present for the examination	
· Lengthy process – the current process was lengthy for applicants	
· Inadequate communication to candidates – need to clarify requirements and expectations to candidates	
· Resource intensive – an individualised process necessarily involves a high level of work	
· Limited assessment of clinical skills - no evidence required of the candidate interacting with clients	
· Lack of a bridging program – no specific programs to assist candidates develop their expertise	
· Limited examination preparation – candidates have no opportunity to prepare for the written examination and this especially disadvantages experienced practitioners who have been out of the education environment for some time. Candidates who are in Australia waiting for the examination have no opportunity to practise their skills	
· Lack of information on performance of overseas-trained practitioners – no feedback on critical areas that overseas-trained professionals struggle with in practice to inform examination content	
· No professional body membership required – inability to monitor professionals or those who were assessed in the past	
· Relevance of accredited courses – courses need to be regularly reviewed, especially with professions in constant change	

**Table 5:** **Possible changes to theassessment processes**

	
· Offering restricted registration – rather than rejecting all applicants who do not meet the full criteria, a partial or limited membership (such as an academic membership) might be offered	
· Adding a formal examination – relying solely on a portfolio or desktop-style assessment is ‘not objective enough in the assessment’	
· Offering off-shore/internet based assessment – to address the issue of candidates needing to come to Australia the possibility of assessing candidates’ clinical skills overseas. Some options were suggested, including Australian assessors going overseas (dependant on candidate numbers) to work with overseas assessors and/or webcam-based assessment	
· Dealing with borderline fail – concern was voiced over borderline fail candidates and it was suggested that there needed to be an option for borderline candidates, as identified by the examiners, to be able to ‘have some provision for perhaps, just make up the work’	
· Training for candidates – the lack of candidate training and/or courses was a common issue raised by professional bodies. Suggested content for such courses included cultural competency, communication skills, knowledge of the Australian health system and or upgrading skills. Issues with training candidates included the financial cost, low candidate numbers, candidates being overseas, Australian professional development courses only being open to Australian registered practitioners and professional bodies not necessarily being educational bodies so courses would need to be outsourced	
· Assessment types – changing or modifying the types of assessment utilised	
· New - for some professional bodies who relied solely on desktop/portfolio there was a feeling that a skills-based assessment was also needed that might be ‘a mixture of both’ [written and clinical]	
· More – a professional body who conducted both written and clinical examinations felt that this was not sufficient and was considering further assessment as they felt that there was not sufficient time to assess all they wished to	
· Technology – consideration was being given to including technology-based clinical assessment (e.g., models, computer imaging) to address issues around using real patients in examinations. One body was experimenting with computerised adaptive testing in which the number and type of items presented was determined by candidate performance	
· Efficiency – consideration was being given to changing the type of clinical examination from a standardised patient model to a clinical supervision model due to resource efficiency issues. Another option was to outsource the examination to educational institutions, although one professional body who did this was considering taking control back	
· Assessors – some professional bodies were making changes to improve the training of their assessors including the development and use of assessor handbooks	

### Examining (Theme 3)

**Figure 3:**
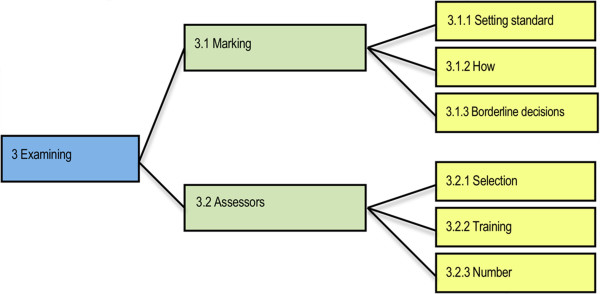
Sub-themes identified within the Examining theme.

Commonly the minimum standard (Theme 3.1.1, Figure 3) required of candidates was that of an entry-level graduate who became eligible to register as a practitioner in Australia by completing the necessary education and clinical requirements. One professional body explained the reasoning behind this decision:

"So the competency basedoccupational standards were developed… all universities areaccredited against those standardsand so all peoplecoming in from overseasagainst those standards."

In specific assessments systems, standards were generally set according to professional standards. One strategy to set criteria in clinical assessment was to use a panel of experts to make decisions. Several professional bodies required candidates to gain a least 75% for their clinical examination with some parts of the assessments being hurdle requirements.

Strategies (Theme 3.1.2) used in marking candidates in clinical examinations included using rubrics with checklists and/or rating scales when assessing candidates. Checklists were used for assessors to note whether or not participants had performed certain components or achieved defined performance indicators. For those that used checklists there was a certain amount of assessor discretion allowed in terms of marking areas that were not included due to the circumstances of the examination.

Assessor judgement was also used when rating. Candidates were marked according to the standard of their performance but an overall pass or fail decision was made at the completion of the clinical assessment. It was noted that this strategy was not based on any statistical grounds and may be reviewed.

*Borderline fail candidates* (Theme 3.1.3) were identified as an issue in assessment by several professional bodies. One strategy used to address borderline candidates was to offer them a supplementary examination. This might mean that the candidate then underwent further testing in a specific area or re-sat the whole clinical examination. Borderline portfolio or desktop assessments were referred to a senior staff member and the application discussed. If a decision could not be reached, it was referred to the assessment committee or another higher-level staff member. On most occasions, if insufficient evidence is found there is an option for candidates to provide further evidence (e.g., complete a specific course) within an extended period rather than re-applying in full.

*Selection of assessors* (Theme 3.2.1) was based on their experience not just in the profession but also in educational assessment as well:

"… again as manypeople did we arerelying heavily on thefact that these peopleare “trained” when theycome to us, trainedby the institutions thatuse them. We thenjust have to reorientthem to the natureof the assessment …"

Assessors from an education background are seen as advantageous not just because of their skills but also because they have experience with entry-level practitioners.

*Training of assessors* (Theme 3.2.2) was noted as an important issue in assessment. Methods of training included formal government-based sessions in recognising fraudulent documents, ‘calibration sessions’ with other assessors to share ideas, working with experienced assessors, handbooks and pre-session briefings. One professional body indicated that reviewing specific assessment cases with assessors had led to more open communication when assessors were unsure of decisions and that this had been highly beneficial. The benefit of a workshop-style approach was highlighted by another organisation because of the opportunity for ideas to be shared. They noted that while rigorous training sessions had worked well for new examiners, established examiners did not find them helpful. They had moved to having new assessors observe the examination, be monitored during their first assessments and then be subject to the same continual auditing as all assessors were in this organisation.

### Cost-efficiency (Theme 4)

**Figure 4:**
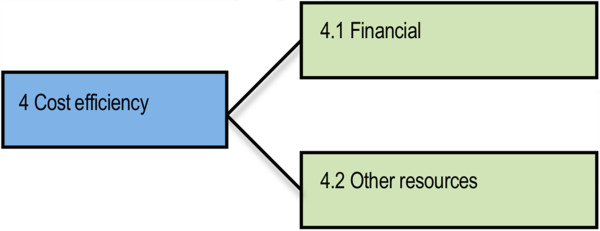
Sub-themes identified within the Cost-efficiency theme.

The cost of examinations (Figure 4) could vary from year to year based on the demand for assessment. Several professional bodies indicated that the number of applications was affected by issues such as the world economic situation and/or movement to another assessment system, such as in New Zealand.

The cost of assessment of overseas-trained practitioners was a significant part of the annual budget for most professional bodies. Although candidate fees contributed to the costs, in most organisations the profession subsidised the costs; the institution running the examinations was ‘extremely kind to us and don’t actually charge us, you know, the full amount’ and/or many professionals voluntarily gave their time. Fees were set at a level that made the examination feasible but not too expensive for candidates.

It was also important for examinations to be efficient in use of other resources. For large candidate numbers, written examinations using MCQs met this criterion. For most professional bodies, however, clinical assessment was resource intensive. A major human resource consideration was the administrative staff that worked to organise examinations and disseminate information to candidates. Organisations considering the introduction of examinations were concerned about the resources required to do this.

## Discussion

The purpose of the present study was to investigate the methods of assessment of overseas-trained health professionals who wish to practice in Australia. The main themes identified in the analysis of responses from the interviews of Australian assessment bodies were *Assessing*, *Processes*, *Examining* and *Cost-efficiency*. Within each theme, multiple levels of sub-themes were also identified.

### Assessing

Under the theme *Assessing* the main sub-themes related to types of assessment and risk management. Assessment bodies use a range of assessment tools when assessing fitness-to-practice, something which in the Australian medical profession is consistent with other countries [8]. In the initial stages, these bodies utilised a desktop assessment process to either screen candidates (ensuring they meet the standard to sit the examination process) or as the sole assessment process. Multiple assessment methods were used to ensure content validity and ensure that candidates were assessed on all competencies and capabilities deemed to be important and relevant for that profession. It may also be that, as Finucane et al. [8] suggest, there is no single assessment method that is suitable to assess fitness-to-practice. Given that it is a high-stakes assessment, decisions about fitness-to-practice should be based on a multitude of information sources [26].

The assessment methods employed by these organisations ranged from short-answer questions and MCQs (testing basic science and theoretical knowledge) [27,28] to the OSCE [15,29] and long case assessment [27,30] (for assessment of clinical capability). In addition portfolio assessments [31] were increasingly used to assess candidates, and a number of organisations indicated that this is an assessment method that may be used at a later date.

Not surprisingly, risk management was a primary concern for most organisations, where different types of risk and associated risk mitigation strategies were discussed. Risks were identified in a number of areas including minimisation of harm to the community and decreasing the risk of harm to patients (or standardised patients) during the examination process. Ensuring that candidates were equipped to cope with workplace based assessments and the environment in which these assessments are conducted was a further concern.

When designing assessments, the bodies identified a number of areas that presented challenges. These included the assessment of cultural competence, communication, knowledge of the Australian health system and after-care/follow-up of the patient. Both cultural competence [32,33] and communication [34,35] have been previously identified within the medical and health education literature as areas that are difficult to assess in a reliable and valid way. In relation to communication, Tamblyn et al. [19] suggest that a cut-off or minimum score be set for communication components of the assessment process in an effort to reduce the number of complaints to professional regulatory bodies. In addition, whilst the organisations interviewed did recognise the importance of cultural competency assessment, particularly related to indigenous Australians, many were only just incorporating, or anticipating incorporating, this area into the assessment processes the organisation used.

### Processes

Under *Processes* the main sub-themes were procedures for conducting assessment, appeal processes, review processes and reflection on the strengths and weaknesses of their systems. Importantly, most organisations reported that their assessment processes and examiners were being continually reviewed. Information about the assessment process, including sample questions, marking criteria etc. were provided to the candidate prior to the assessment process. Written assessments were largely undertaken off-shore, that is, not in Australia. Caution was advised when running examinations off-shore as it was a complex task to have the correct candidate at the correct site sitting the correct examination and receiving the correct results. The organisational difficulty was increased with larger numbers of candidates.

Post-assessment issues including candidate feedback and appeals were also canvassed. Most organisations provided feedback to candidates who failed an element(s) of the assessment process and this assisted in clarifying areas in which the candidate needs to improve and also minimise appeals, ensuring the process is fair and transparent [8]. Organisations were understandably keen on minimising appeals, and in most cases appeals were only available to the candidate if an examination process issue was identified. These steps to minimise appeals would also have an impact on making the assessment defensible from a legal standpoint. Although this was not articulated by participants in the current study, previous research has indicated this is a concern for such organisations [8], and minimised by the use of valid and reliable assessment strategies. When asked to reflect on their processes, organisations identified numerous strengths and weaknesses, and these organisations also presented planned or potential changes to their assessment processes.

### Examining

Within the theme *Examining*, there were two sub-themes related to marking (including processes for those who fail) and assessors (selection and training). Marking was undertaken using checklists [36], ensuring that candidates performed required elements, however there was little discussion of the use of global or holistic assessments. The use of holistic assessments is becoming widely reported in the literature as a valid and reliable assessment outcome [37-40], although it appears that this has yet to make its way into the assessments undertaken by the organisations. The use of global assessments has been demonstrated to improve the reliability and validity of an assessment, particularly where communication skills are assessed [41,42].

Most organisations spent large amounts of time and money on their examiners, in terms of training, recruitment and payments to assess. Examiners were typically selected based on clinical experience as well as experience in education [43-45], which ensures that it is a peer assessment process [8]. Formal training sessions were often undertaken, and new assessors were paired with more experienced assessors, to aid in their development. Examiner training is widely accepted to improve the reliability of an assessment as well as improve examiner confidence in the assessment process [43,46-49]. All examiners were the subject of ongoing auditing and assessment, and therefore “…remain competent in what they do” [8]. Where organisations did not have formal examiner training processes in place, it was anticipated that they were to implement a process in the near future.

### Cost-efficiency

The range of practices reported under the theme *Cost-efficiency* was relatively limited. The size of the organisation had an impact on the financial elements of the assessment process; large organisations were able to make money on their examinations and use this to further develop their processes, smaller organisations often charged candidates the ‘cost’ of conducting the assessment, leaving them with very little in the way of financial resources to develop their assessment processes. Whilst clinical assessments were labour and cost intensive, particularly in relation to the administration of the assessment, organisations did not perceive this to be a major issue.

## Conclusions

Most of the organisations who participated in the current study have invested large amounts of time and resources, both financial and administrative, into the development and ongoing review of their assessment processes. Whilst many organisations are utilising assessment methods they have employed for a number of years, there was recognition that ‘newer’ assessment types such as the portfolio may be useful in the overall assessment process. The assessment methods were often chosen based on the resources available to that organisation (i.e. MCQ for medicine). Most processes include multiple assessment methods, with these methods blueprinted to assess a range of competencies and capabilities for that profession. Overall, the organisations interviewed provided an impression of the use of the literature to inform their assessment processes and the use of robust, defensible assessments of overseas-trained health professionals who wish to practice in Australia.

## Competing interests

The authors declare that they have no competing interests.

## Authors’ contributions

All authors were involved in the design of the study. VS and BV undertook the literature review. VS lead the focus groups and interviews. MW assisted with the focus groups and interviews. RG, CG, VS and BV analysed the data. GF and PMcL developed the discussion. All authors contributed to the compilation and review of the manuscript. All authors read and approved the final manuscript.

## Pre-publication history

The pre-publication history for this paper can be accessed here:

http://www.biomedcentral.com/1472-6920/12/91/prepub
